# Design of Examination Management System for Engineering Management

**DOI:** 10.1088/1755-1315/474/7/072047

**Published:** 2020-04

**Authors:** Hualong Cai, Yong Yao, Jingwen Cai

**Affiliations:** 1 College of Civil Engineering, Jiaying University, Meizhou, Guangdong, 514015, China; 2 Dongguan Sino-German Technical School, Dongguan, Guangdong, 523000, Chinawhucad@whu.edu.cn

## Abstract

Through the analysis of the engineering management training system, we know that the courses in the system can be divided into theoretical, practical and experimental courses. We aims to develop an examination management system by restructuring the evaluation process of the three types of courses. And the organizational structure, hardware architecture, and software architecture of the test management system are designed to be applied in the entire process of system development, operation and maintenance. Besides, under the influence of “the pneumonia caused by novel coronavirus, COVID-19” on education, we wish that the examination management system is more suitable for the needs of the macro environment, and the results can be used to the training and evaluation of other professionals.

## 1. Research Background

At present, "the pneumonia caused by novel coronavirus, COVID-19" has a big effect on education. Teaching and learning rely more on the Internet platform. With the update of the scientific system, more emerging disciplines are needed to support the rapid development of society, and the engineering management major is also constantly renewing its own teaching management and the evaluation models of student's learning.[1] In order to cultivate diversified and innovative engineering talents, the engineering management major applies the concept of adapting to changes and shaping the future and takes inheriting and innovating, crossing and integrating, coordinating and sharing as the main approaches, with the features of strategic, innovative, systematic and open. The construction of disciplines, a systematic project, leads to the adjustment of the original discipline system, and to the updating of the supporting evaluation system as well. With the aims of adapting to the content adjustment of the discipline and meeting the needs of detailed and quantified dynamic evaluation of student learning quality, it is very necessary to improve existing the methods of examination management and constructing a new test management system. The article takes the engineering management specialty training system as the background, designs and studies the supplementary examination management system with the supports the new form of requirements.

## 2. Course type classification

The engineering management specialization involves the fields of civil engineering and management science and engineering. According to the teaching methods, the courses are mainly divided into theoretical courses, practical courses, and professional experimental courses, as shown in Table 1.

Content from this work may be used under the terms of the Creative Commons Attribution 3.0 licence. Any further distribution of this work must maintain attribution to the author(s) and the title of the work, journal citation and DOI.
Published under licence by IOP Publishing Ltd

Table 1. Course type and content

| | Course type | Course classification and content |
|---|---|---|
| 1 | Theoretical courses | Advanced mathematics, engineering mechanics, basics of financial accounting, engineering cost management, etc. |
| 2 | Practical courses | Engineering surveying practice, engineering budget course design, etc. |
| 3 | Experimental courses | civil engineer materials experiment |

## 3. Course evaluation process

### *3.1 Theory course evaluation process*

The title is set 17 point Times Bold, flush left, unjustified. The first letter of the title should be capitalized with the rest in lower case. It should not be indented. Leave 28 mm of space above the title and 10 mm after the title. The theory course not only assesses the students 'basic knowledge, but also improves the students' autonomous learning ability. The course evaluation includes objective questions, subjective questions and course thesis [2]. The score ratio of each part is determined according to the course content. The operation steps are as follows, and the process is shown in Figure 1.

Step 1: the head of the department introduces the course setting plan of the engineering management specialty program into the system to form a directory tree of examination courses, and each course corresponds to credits and hours.

Step 2: Students swipe a card for identity authentication, log in to the system, and operate in order. The order is selecting courses, objective questions, subjective questions and course papers. Each type of question can be tested separately, and completing the test at a time is not necessary.

Step 3: ①After the candidates select objective questions, questions with limited score will be choosed randomly from the question bank. Then, the candidates answer the questions alone on a fixed computer and submit the test paper within the required time, obtaining a score. If the candidates are satisfied with the score, he can get the score as “grade one”, and the database cannot be changed any more. The objective question allows the candidates to take the test twice and choose a higher score as the final score if they are not satisfied with the first score. ②After the candidates select subjective questions, questions with limited score will be choosed randomly from the question bank. The test center staff assigns a fixed seat for them to answer the question after the candidates print the question. Finishing within a limited time, the candidates submit the test paper to the test center staff. Then, the test center staff scans the test sheet into the database, and the teacher can mark it through the network because the examination barcode corresponds to the candidate.③ After students choose a course paper, they submit their paper file and upload it to the database, and the teacher can review and mark through the network.

Step 4: Three parts' scores are converted into percentage points according to a predefined ratio, which becomes the students' final grade for the course.

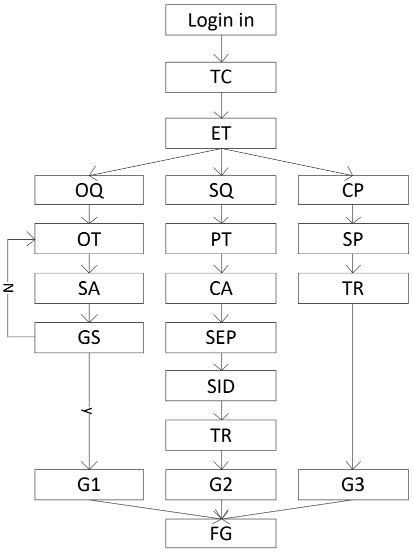

ET: Examination Type
OT: Online Test
SA: Submit Answer
GS: Grade Save
SQ: Subjective Questions
PT: Print test Papers
CA: Closed Answer
CP: Course papers
SP: Submit a paper
G1: Grade part one
G2:Grade part two
G3:Grade part two
FG: Final Grade
TC: Theoretical Courses
SEP: Submit Examination Papers
OQ: Objective Question
SID: Scan Into the Database
TR: Teachers review the papers

Figure 1. Theory course flow

*3.2Practical course evaluation process*

The practical course should not only examine the students' ability to compile a practical plan, the ability to write a practical report, but also the practical ability to practice. Practice evaluation is divided into video recording of practice process and preparation of practice report. The score ratio of each part is determined according to the course content. The operation steps are as follows, and the process is shown in Figure 2.

Step 1: Candidates log in to the system on the computer, select practical courses and submit practical scheme.

Step 2: Candidates should complete the practice process and submit videos of key practice processes.

Step 3: Candidates should finish the practice report and submit the practice report.

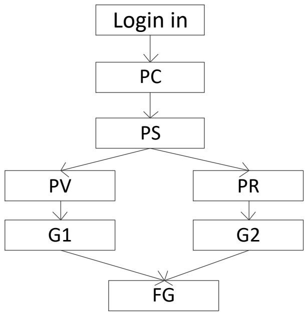

PC: practice course
PV: practice video
G1：grade part one
FG: final grade
PS: practice scheme
PR: practice report
G2：grade part two

Figure 2. Practical course flow

*3.3Experimental course evaluation process*

The experimental course should not only examine the students' ability to prepare experimental schemes, the ability to write experimental reports, but also the practical ability to experiment. The experimental evaluation is divided into video recording of experimental process and preparation of experimental report. The score ratio of each part is determined according to the course content. The operation steps are as follows, and the process is shown in Figure 3.

Step 1: Candidates log in to the system on the computer, select experimental courses and submit experimental schemes.

Step 2: Candidates should complete the experimental operation and submit a video of the experimental process.

Step 3: Candidates should finish the experiment report and submit the experiment report.

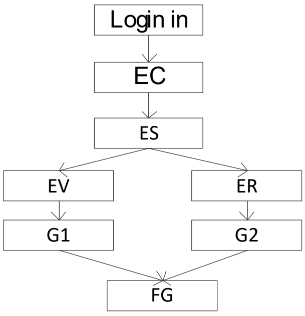

EC: experiment course
EV: experiment video
G1：grade part one
FG: final grade
ES: experiment scheme
ER: experiment report
G2：grade part two

Figure 3. Experiment course flow

## 4. System architecture design

### *4.1 System organization structure*

Changes in evaluation methods will surely lead to changes in the evaluation organization system. A separate test center will be set up to operate and maintain the test management system. For theoretical courses, students can go to the test center at any time to choose their own relevant subjects and examination types. For practical and experimental courses, students can enter the system platform via the Internet, submitting relevant results on personal computers. Teachers can review the results submitted by students through the Internet and grade them.

### *4.2System hardware architecture*

The system builds a hardware architecture. According to the number of users, multiple servers need to be set up for web services, database services, application services, and backup storage [3]. For the Internet connection, setting up a firewall to ensure system security is necessary. At the test center, a switch should be set up to connect the examination and management computers in the test room. As shown in Figure 4.

In the intranet, students can complete the objective and subjective test evaluations of theoretical courses in the test center. In the Internet, students can submit evaluation results of practical courses and experimental courses through their own computers. Teachers can grade students' submitted course results, both in the intranet and extranet.

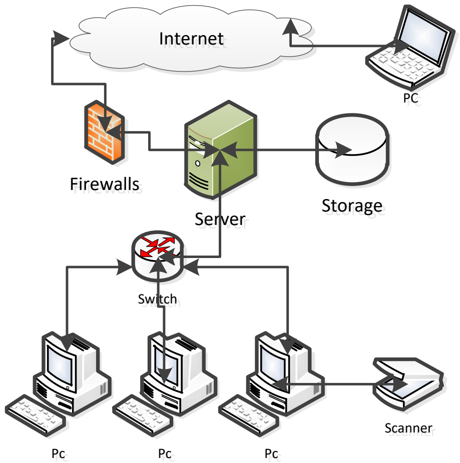

Figure 4. System hardware architecture

### *4.3 System software architecture*

The system software is divided into three layers of architecture: presentation layer, business logic layer and data access layer [4], as shown in Figure 5.

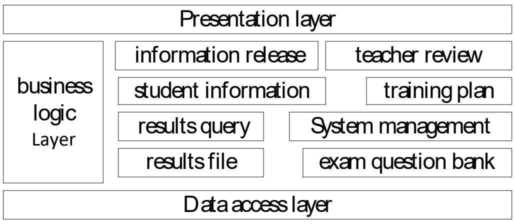

Figure 5. System software architecture

Examination information release: You can publish dynamic information about the test center.

System background management: The system administrator logs in to configure the permissions and functions of the system.

Student information module: the functions of adding, deleting, checking and changing students' information.

Training program module: The head of the department can introduce the learning and examination courses required for each student training into the system.

Student results query: Students can query their results, comprehensive rankings and other information.

Teacher review module: Teachers can evaluate and grade students' submissions.

Student grade files: The grade files of students can be managed and backuped.

Examination question bank management: The exam question bankup should be updated, replaced, etc.

## 5. Conclusion and Outlook

The regeneration of the examination process and rational system architecture design can improve the efficiency of engineering management professionals evaluation. The application of the examination management system can standardize the evaluation system of engineering management professional courses. The means of "promoting learning through examinations" can surely improve students' learning effects. In addition, under the current " the pneumonia caused by novel coronavirus, COVID-19 " and the Internet of teaching, the examination management system can better meet the needs of the situation. The test management system solidifies the training management model and can be applied in other majors as well.

## Acknowledgments

The research was supported by Jiaying University's Teaching Reform Research Fund (JYJG20180108).
